# Mortality rate of people exposed to Mustard Gas during Iran-Iraq war in Sardasht, Iran: a 32 years retrospective cohort study

**DOI:** 10.1186/s12889-022-13520-2

**Published:** 2022-06-09

**Authors:** Mohammad Hasan Rabiee, Mostafa Ghanei, Hossein Amini, Aliasghar Akhlaghi

**Affiliations:** 1 Department of Epidemiology, Faculty of Veterinary Medicine, University of Tehran, Tehran, Iran; 2 Chemical Injuries Center, Systems Biology and Poisoning Institute, Baqiyatallah University of Medical Sciences, Tehran, Iran; 3 Department of Epidemiology, School of Public Health, Iran University of Medical Sciences, Tehran, Iran; 4 Department of Epidemiology and Reproductive Health, Reproductive Epidemiology Research Center, Royan Institute For Reproductive Biomedicine, Academic Center for Education, Culture and Research, Tehran, Iran

**Keywords:** Mortality, Mustard gas, Sardasht, Iran

## Abstract

**Background:**

Mustard gas (MG) is one of the most widely used chemical weapons in the past century. However, little information exists concerning long-term mortality from MG exposure. In this study, we investigated mortality rate among civilian people exposed to MG during Iran-Iraq war in Sardasht in Iran after 32 years.

**Methods:**

In this retrospective cohort study, data of people exposed to MG in Sardasht in 1987 were extracted from the Veterans and Martyr Affair Foundation of Iran up to March 20, 2019. Mortality rate, cumulative mortality and standardized mortality ratio with 95% confidence interval were calculated to explain mortality in the cohort, and then compared with general Iranian population. Cox regression analysis was used to indicate factor affecting the risk of death in the cohort.

**Results:**

Out of 1,203 exposed people at the beginning of the period, 148 people died by the end of the study, with an average age of 66.42 at the time of death. Total person-years of the people up to end of the study were 38,198.63 and mortality rate was equal to 387 per 100,000 persons-years. Total number of observed deaths was less than expected death and the all-cause standardized mortality ratio (SMR) was determined as 0.680 (95% CI: 0.574 – 0.798). Cause-specific SMR showed that observed death due to respiratory diseases was higher than expected (SMR: 1.75) (95% CI: 1.145 – 2.569). The results of univariate and multivariate cox regression analysis showed that increasing age and having severe late complications in lung were associated with increased risk of death among people in the cohort.

**Conclusion:**

In general, this result indicated that acute exposure to MG, even without wearing protective clothing and masks, could not increase all-cause mortality after 32 years if accompanied by special and ongoing care for those exposed.

## Background

Mustard agents are vesicant chemicals agent, including two important types: Sulfur mustard and Nitrogen mustard. Sulfur mustard, also known as Mustard gas (MG), is the most well-known and hazardous vesicant chemicals agent which was synthesized for the first time by Despretz in 1822 [1, 2]. This fast-absorbing substance can enter the body through various routes including the respiratory tract, skin, and anterior surface of the eye, as well as gastrointestinal tract, which consequently can lead to recurrent complications in various organs and death in the short and long term [3–5].

The use of MG as a chemical weapon in war has so far caused hundreds of thousands of people to be exposed to small or large amounts of this agent in acute form. In other words, since the first use of MG as a chemical weapon by the German army in 1917 in the war against Canadian and French troops, this agent has been repeatedly used in various wars against different countries such as Iran in the 8-years war between Iran and Iraq which has caused injury, complications and death to military and civilian people [6, 7].

Sardasht is one of the cities in Iran that its civilians were exposed to MG during the Iran-Iraq war from 1981 to 1989. The city, located in northwestern Iran with latitude of 36º 9′ 4.48″ and a longitude of 45º 29′ 10.46″, was bombarded by Iraq on June 28, 1987. The bombing dropped seven 250 kg MG bombs, four of which exploded in a densely populated downtown, affecting civilians [3]. People exposed to MG in the city of Sardasht in 1987 during the Iran-Iraq war were civilians and had similar characteristics to the general population. In addition, these people were exposed to this agent without any protective clothing and mask.

So far, a number of studies have investigated the long-term mortality rate of exposed people to MG in war; however, in one hand, their number is limited and, on the other hand, they are all conducted on a military population and there is no information on long-term mortality in a civil population such as Sardasht [4, 8]. Meanwhile, after 32 years of exposure of these people to this agent in Sardasht, no study has measured the impact of exposure to MG on mortality rate in these people. Therefore, we investigated mortality rate among people exposed to MG during Iran-Iraq war in Sardasht, Iran after 32 years. It should be noted that the paper does not address other long-term medical consequences of exposure to the agent.

## Methods

### Study design and study population

In this retrospective (historical) cohort study, the study population included people exposed to MG in Sardasht in 1987 based on the approval of the Veterans and Martyr Affair Foundation (VMAF). VMAF is a legal authority in Iran that provides pension and health services to war survivor. This foundation registers all survivors of war and then regularly provides long-term care services to them and their families by maintaining excellence in health care services through professional collaboration [7, 9]. Therefore, the inclusion criterion was the approval of the VMAF of Iran to contact of the persons with the chemical agent of MG and the exclusion criterion included the occurrence of premature death. In this study, premature death refers to death at the time of exposure (during the first 14 days after exposure).

This study was approved by review board of Biotechnology Development Council (BIODC) of Iran. No informed consent was needed, according to the regulations approved by the Iran national committee for ethics in biomedical research, as it uses non-identifiable data from an existing data set [20].

### Data collection

Data related to health status (death/life) and other information of the subjects were obtained by referring to the VMAF of Iran and reviewing the VMAF database up to March 20, 2019. Normally, all the information related to the deaths of war survivors in Iran was transferred to the VMAF of Iran, where a death commission consisting of various specialists and experts investigates the causes of death. The members of this committee are aware of the possible chronic effects of MG and determine the causes of death based on the medical record and death certificate.

In addition to data regarding health status (death/life) of the subjects, other information including gender (male/female), age of the person at the time of exposure, severity of late complications (no symptom, mild symptom, moderate symptom, severe symptom), organ of the late complications (lung, eyes, skin), evacuation and hospital admission status (EA, NEA, None) were also taken from the mentioned database and then recorded. In order to classify subjects according to evacuation and hospital admission, based on the evidence of exposure to MG, the data were categorized into 3 groups: (1) evacuated and admitted (EA), confirmed history of exposure in the affected geographic region with evacuation and hospital admission; (2) not evacuated or admitted (NEA), confirmed history of exposure without evacuation or hospital admission; and (3) undocumented cases, not classified in the other 2 groups but showing late clinical manifestations suggestive of exposure. The classification criterion for the severity of the late complications which periodically examined and record by a specialized medical team was based on the protocol compiled by a panel of experts at the VMAF of Iran. Based on this protocol, the complications in these people were divided into four categories: no symptoms, mild, moderate and severe symptoms. The panel included pulmonologists, psychiatrists, neurologists, ophthalmologists, dermatologists, cardiologists, forensic specialists and nephrologists who reviewed the relevant documents and evidence and categorized them [7]. Definitions of late complications in lung, skin and eye are as follow:

- Lungs:
  Categorization of complications in lung following MG exposure were defined using spirometry results of forced expiratory volume in the first second (FEV1) as follows: no symptoms, FEV1 higher than 80%; mild, FEV1 higher than 70% but lower than or equal to 80%; moderate, FEV1 higher than 50% but lower than or equal to 70%; and severe, FEV1 lower than or equal to 50% with normal arterial blood gas or severe tracheabronchomalacia [7, 10].
- Skin:
  Category of skin complications in MG exposed victims were determined as follows: Mild category was defined as the presence of an MG-specific scar (pigmentation, vascular, or trophic changes) across less than 5% of the body surface; the presence of such a scar plus mild dermatitis and mild xerosis or mild-to-moderate autoimmune diseases; and concurrent medical documents plus severe autoimmune diseases or any mild dermatitis. The moderate category was also defined as the presence of an MG-specific scar affecting 5-20% of the body surface; presence of an MG-specific scar with significant trophic changes in the genital or anus areas; the presence of an MG-specific scar of any size with or without concurrent medical documents plus severe dermatitis covering less than 50% of the body surface; concurrent medical documents with severe dermatitis or xerosis covering less than 50% of the body surface; and the presence of an MG-specific scar of any size with or without concurrent medical documents plus any severe autoimmune disease affecting the skin covering more than 50% of the body surface. The severe category included an MG-specific scar covering more than 20% of the body surface; the presence of an MG-specific scar of any size with or without concurrent medical documents with severe dermatitis or xerosis covering more than 50% of the body surface; and concurrent medical documents with severe dermatitis or xerosis covering less than 50% of the body surface [7, 11].
- Eyes:
  Complications in eye following MG exposure were categorized as follows: Mild category was defined as burning, itching, tearing, redness, foreign body sensation, blurred vision, conjunctivitis, subconjunctival hemorrhage, photophobia, or conjunctival vascular changes (telangiectasia and vascular changes, edema of the eyelid, papillary changes) of 1 or both eyes. The moderate category was defined as the same changes in the mild category along with conjunctival ischemia in the limbus area, as well as unilateral or bilateral closure or cauterization of punctum. The severe category was: those observations in the moderate category along with symptoms of corneal involvement, epithelial and subepithelial opacity and anterior stroma in the cornea, keratopathy, pannus, hyperpigmentation around the nerve, iron deposition in the cornea and corneal vascularization, stenosis, and involvement of less than half unilaterally or bilaterally in both eyes [7, 12].

### Statistical analysis

After collecting data, all statistical analyses were performed in Stata software version 15. Initially, the frequency and relative frequency index were used to express the basic characteristics of the subjects.

In order to describe the mortality in the cohort, we used cumulative mortality rate and mortality rate index. To calculate mortality rate index, we first calculated the person-years at risk in these individuals from the time of exposure to MG to the occurrence of death in the case of subject who died, or at the end of follow up for other subject. Then all-cause mortality rate and the cause-specific mortality rate were calculated.

In order to compare the mortality in the cohort with the general population (Iran), we used standardized mortality ratio (SMR) index. SMR is the number of observed deaths divided by the expected death. To calculate expected death, first, mortality rate of the general population according to sex, age (5 year age group) and calendar year were obtained using the information published by GBD 2019 Demographics Collaborators [13]. Then, expected death was determined by multiplying the mortality rate of the general population and the person-years of the cohort. Finally, we calculated all-cause SMR and cause-specific SMR. Also, all-cause SMR was determined according to age, sex and severity of late complications. For each SMR, a 95% confidence interval was calculated, assuming that the number of deaths observed follows the Poisson distribution.

Eventually, cox regression model was used to determine the relationship between the independent variables (age at the time of exposure, sex, severity of late complications, evacuation and hospital admission status) and risk of death. To do this, first, the univariate Cox regression model was applied and the variables that had a significance level of less than 0.1 in this model were included in the multivariate Cox regression model. Finally, variables that had a significance level of less than 0.05 in the multiple models were considered as a factor affecting the risk of death in the cohort.

## Results

### Basic characteristics of people exposed to MG in Sardasht

The results of this study showed that most of the cohort subject (85.7%) were under 40 years old at the time of exposure. The average age of people at the time of exposure was 25.51. The results also demonstrated that most of the subjects were male (60.5%) and 39.5% were female. The full results of basic characteristic of the subject including age at the time of exposure, sex, severity of late complications, as well as evacuation and hospital admission status were presented in Table 1.

**Table 1:** Basic characteristics of people exposed to MG in Sardasht, Iran

				Frequency	Relative frequency
Age at exposure time		Under 20		487	40.5
		20–40		543	45.1
		Above 40		173	14.4
Sex		Male		728	60.5
		Female		475	39.5
Severity of late complications in different organ	Lung	No symptom		378	31.4
		Mild		633	52.6
		Moderate		169	14
		Severe		23	2
	Skin	No symptom		916	76.1
		Mild		238	19.8
		Moderate		43	3.6
		Severe		6	0.5
	Eye	No symptom		994	82.6
		Mild		198	16.5
		Moderate		10	0.8
		Severe		1	0.1
Evacuation and hospital admission status		EA		416	34.6
		NEA		787	65.4
Current health status		Alive		1055	87.69
		Dead	Known cause	76	6.32
			Unknown cause	72	5.99

### Mortality of people exposed to MG in Sardasht and its comparison with the general population

The results of the study concerning mortality in the subjects from June 28, 1987 to March 20, 2019 showed that the total cumulative mortality rate during this period was equal to 12.30%. In other words, among the people who were exposed to MG at the beginning of the study, 12.30% of them (1203/148) died by the end of the study. Moreover, mortality rate between these years was found to be 387 per 100,000 person-years. In other words, 148 deaths per 38,198.63 person-years occurred in this study. The average age of the died people at the time of death was 66.42. In addition, the results showed that out of these 148 deaths, 72 had unknown causes and the cause of death of 76 people was determined, among which the most causes belonged to cardiovascular diseases (33 cases) and respiratory diseases (26 cases). The complete results concerning mortality in the subject is shown in Table 2.

**Table 2:** All-cause and cause-specific SMR among people exposed to MG in Sardasht, Iran

	ICD 10	Observed death	Mortality rate	Cumulative mortality rate	Expected death	SMR	SMR (95% CI)
All cause	A00-Z99	148	0.00387	0.123	217.65	0.680	0.574 – 0.798
Neoplasm	C00-D49	9	0.00023	0.0075	36.70	0.245	0.112 – 0.465
Circulatory system disease	I00-I99	33	0.00086	0.0274	98.65	0.335	0.230 – 0.469
Respiratory system disease	J00-J99	26	0.00068	0.0216	14.83	1.753	1.145 – 2.569
Genitourinary system disease	N00-N99	3	0.000078	0.0025	6.57	0.457	0.094 – 1.334
External cause	S00-Y88	5	0.00038	0.0041	34.12	0.147	0.047 – 0.341

•72 cases with unknown causes are not included in this table

The results of comparing the mortality in the study population with general population (Iran) are shown in Table 3. Based on the results, all-cause standardized mortality ratio index was 0.680. In other words, the expected mortality for the study population was 217.65, while the observed number of deaths in this population was determined 148. Also, the all-cause standardized mortality rate were calculated according to sex, age at the time of exposure, year, and severity of the late complications, as well as evacuation and hospital admission status; furthermore, cause-specific mortality ratio was determined  (Tables 2 and 3). This finding showed that people with severe late lung complications had almost twice as many deaths as expected (SMR: 2.371) and people with severe late skin complications were also more likely to die than expected (SMR: 1.098). In addition, cause-specific standardized mortality ratio showed that observed death due to respiratory diseases was higher than expected (SMR: 1.75).

**Table 3:** All-cause SMR among people exposed to MG in Sardasht, Iran according to independent variable

			Person-years	Observed death	Expected death	SMR	SMR (95% CI)
Total			38,198.63	148	217.65	0.68	0.574 – 0.798
Age at exposure time		Under 20	15,838.86	12	22.78	0.526	0.272 – 0.922
		20–40	17,417.25	54	65.35	0.826	0.621 – 01.079
		Above 40	4942.52	82	129.5	0.633	0.503 – 0.786
Sex		Male	15,262.03	45	62.75	0.717	0.523 – 0.959
		Female	22,936.6	103	154.9	0.665	0.542 – 0.806
Years		1987—1990	4219.68	0	15.94	0	0 – 0.231
		1991—1995	6024.19	0	20.49	0	0 – 0.180
		1996—2000	6020.38	9	29.01	0.31	0.141 – 0.588
		2001—2005	6016.19	20	32.35	0.618	0.377 – 0.954
		2006—2010	6007.68	39	39.9	0.977	0.695 – 1.336
		2011—2015	6006.68	43	45.44	0.946	0.684 – 1.274
		2016 -2019	3903.83	36	34.53	1.042	0.730 – 1.443
Years	Lung	No symptom	12,040.78	41	60.99	0.672	0.483 – 0.913
		Mild	20,238.39	64	112.66	0.568	0.437 – 0.726
		Moderate	5305.31	31	38.94	0.796	1.133 – 0.542
		Severe	614.15	12	5.06	2.371	4.250 – 1.257
	Skin	No symptom	29,166.51	105	152.18	0.689	0.564 – 0.835
		Mild	7496.99	35	56.54	0.619	0.431 – 0.862
		Moderate	1339.96	7	8.01	0.873	1.823 – 0.355
		Severe	195.17	1	0.91	1.098	6.916 – 0.031
	Eye	No symptom	31,574.67	117	171.17	0.683	0.565 – 0.819
		Mild	6272.75	30	44.35	0.676	0.457 – 0.968
		Moderate	318.46	1	1.9	0.526	3.158 – 0.014
		Severe	32.75	0	0.23	0	0
Evacuation and hospital admission status		EA	12,969.08	73	96.8	0.754	0.591 – 0.949
		NEA	25,229.55	75	120.85	0.62	0.488 – 0.778

**Fig. 1:**
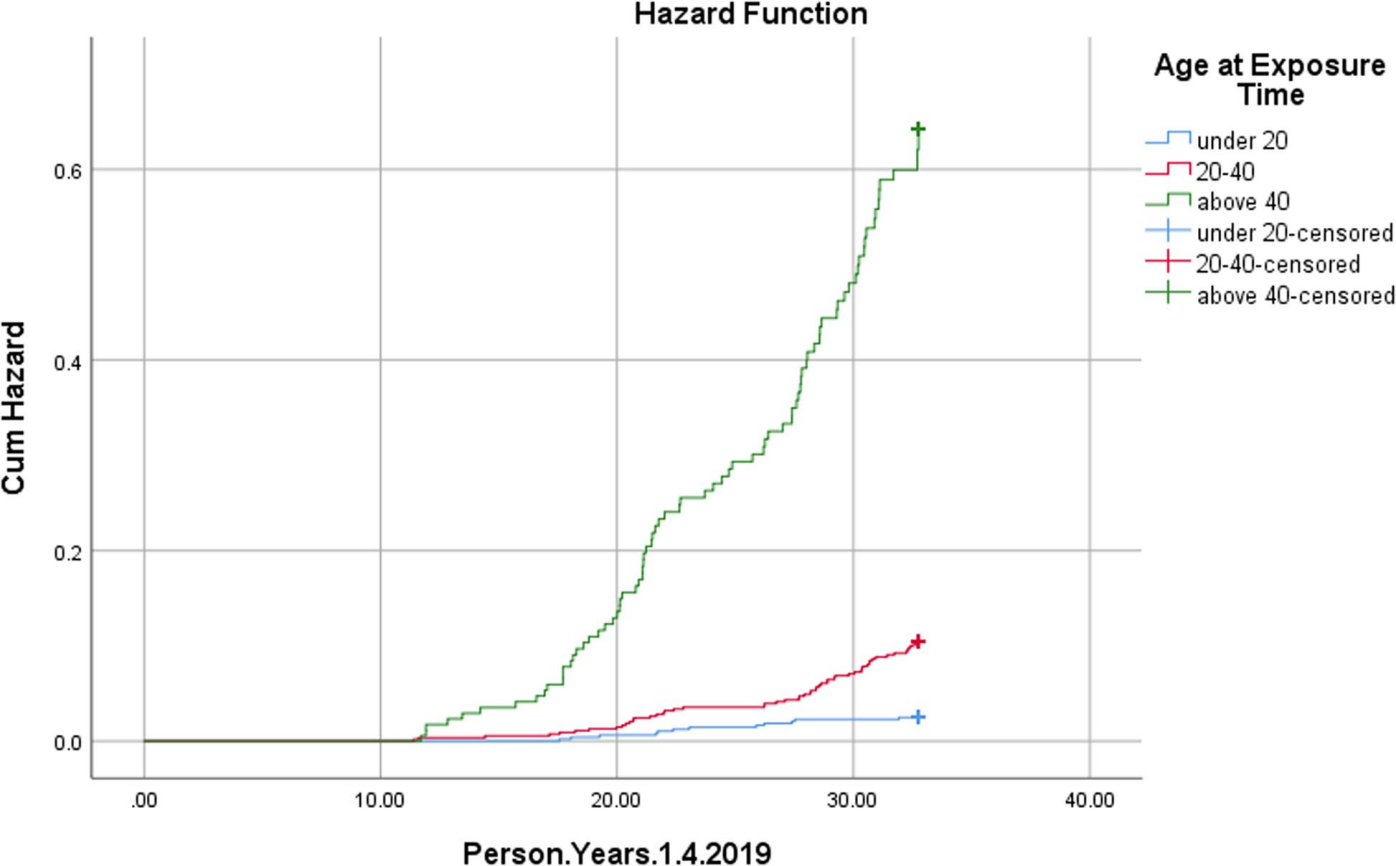
Cumulative death rates in the people exposed to MG in Sardasht, Iran stratified by age

**Fig. 2:**
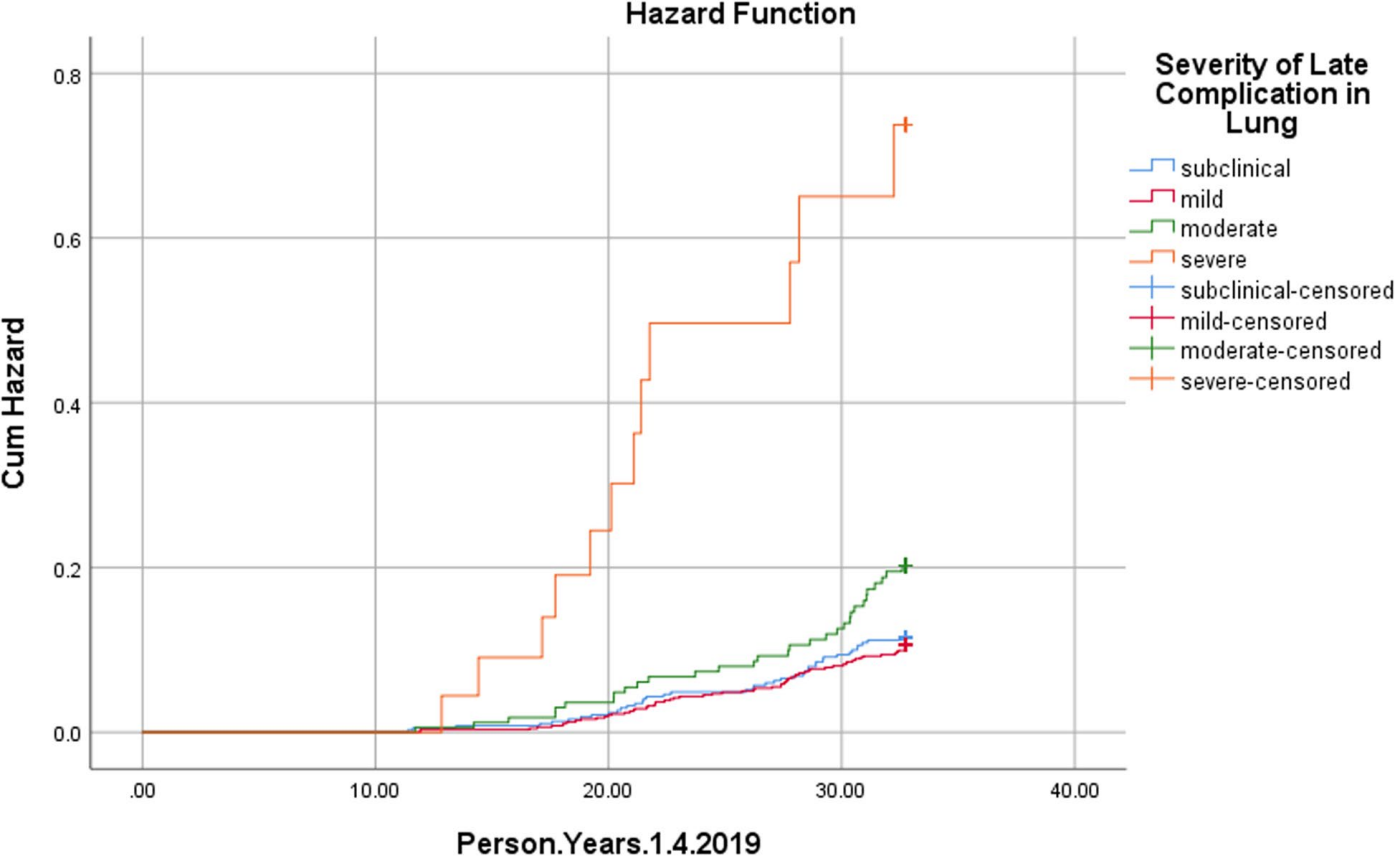
Cumulative death rates in the people exposed to MG in Sardasht, Iran stratified by severity of late complications in lung

The Cox regression analysis was used to determine effect of age at the time of exposure, sex and severity of late complications, as well as evacuation and hospital admission status on mortality in the subjects; the findings showed that sex was not related to the risk of mortality in these individuals, while significant direct relationships of age at the time of exposure (HR: 1.077) (*P* Value: 0.000) and severity of lung complications with the risk of death were found (HR: 5.954) (*P* Value: 0.000). The complete results of Cox regression analysis are presented in Tables 4 and 5. Subsequently, cumulative death rate curves for the variables of age at the time of exposure and severity of lung complications were obtained using Kaplan Meyer analysis method (Figs. 1 and 2).

**Table 4:** Univariate cox proportional hazards regression: factors associated with death in the people exposed to MG in Sardasht, Iran

		B	HR	HR (95% CI)	*P* Value
Sex	Male	0.438	1.549	1.091 – 2.198	0.014
	Female				
Age at exposure time		0.075	1.078	1.066 – 1.086	0.000
Severity of late complications in lung	No symptom				
	Mild	- 0.077	0.925	0.625 – 1.369	0.699
	Moderate	0.556	1.744	1.093 – 2.779	0.019
	Severe	1.954	7.057	3.706 – 13.437	0.000
Severity of late complications in skin	No symptom				
	Mild	0.275	1.316	0.897 – 1.929	0.160
	Moderate	0.391	1.479	0.688 – 3.179	0.316
	Severe	0.332	1.393	0.194 – 9.984	0.741
Severity of late complications in eye	No symptom				
	Mild	0.260	1.297	0.868 – 1.937	0.203
	Moderate	-0.168	0.846	0.118 – 6.054	0.867
	Severe	-	-	-	-
Evacuation and hospital admission status	EA				
	NEA	-0.666	0.514	0.372 – 0.708	0.000

**Table 5:** Multivariate cox proportional hazards regression: factors associated with death in the people exposed to MG in Sardasht, Iran

		B	HR	HR (95% CI)	*P* Value
Sex	Male	0.194	1.214	0.850 – 1.734	0.286
	Female				
Age at exposure time		0.074	1.077	1.066 – 1.087	0.000
Severity of late complications in lung	No symptom				
	Mild	-0.139	0.870	0.572 – 1.322	0.516
	Moderate	0.277	1.319	0.821 – 2.117	0.252
	Severe	1.784	5.954	3.036 – 11.676	0.000
Evacuation and hospital admission status	EA				
	NEA	-0.054	0.947	0.645 – 1.389	0.947

## Discussion

This study aimed at investigating the mortality rate of people exposed to MG in the city of Sardasht, which was bombarded by seven 250 kg MG bombs during the Iran-Iraq war. The results of this study are important because, unlike other similar studies, the exposed individuals in Sardasht are civilians and therefore have similar characteristics to the general population. Moreover, they had no protective clothing and mask to protect themselves from this agent.

The results of this study on all-cause mortality and its comparison with the general Iranian population showed that mortality rate in people exposed to MG in Sardasht did not increase after 32 years. Indeed, observed death was remarkably lower than expected death (SMR: 0.687). Most of the similar studies have focused on the impact of chronic exposure or on the impact of acute exposure on cause-specific mortality. However, in a similar study concerning impact of acute MG exposure on all-cause mortality, Norman et al. selected a sample of 2718 men admitted to American special gas hospitals in August and October of 1918 at the end of the World War I with clear evidence of MG exposure. Then, they compared mortality of the cohort to the general population (US white male population) from 1918 to 1965 and indicated that observed death was very close to expected death after 47 years (SMR: 0.99) [4]. However, in the present study, although the subject were civilians who had no protective clothing and mask to deal with this agent, the number of deaths observed was remarkably lower than expected. As an explanation, we believe that the result of the current study can be partly due to the provision of immediate and special care under the national care management scheme. Another study by Mokida et al. in Japan compared the mortality of people exposed to MG in factory workers to the general Japanese population after 57 years, and they also showed lower number of observed deaths than the number of expected deaths. They attributed the reason to the receipt of special health care beside existence of a healthy worker effect [5]. However, in our study, the role of receiving special health care is much more obvious; the study mentioned in Japan was based on factory workers population, while the current study is conducted on civilians and here we do not have a healthy worker, where we can attribute this reduction in mortality to the role of receiving special health care with more certainty. As a further explanation, other studies have highlighted the provision of good special services for war survivors in Iran. For example, the study by Mousavi et al., indicated that health care utilization (outpatient and inpatient) are more frequent among war survivors than the Iranian general population [14].

Besides, all-cause standardized mortality ratio according to the severity of late complications showed that in individuals with severe lung complications and severe skin complications, the observed death was much higher than expected. Of course, it should be noted that the severe skin complications were slightly more than expected, while the severe lung complications were more than twice the expected amount. Moreover, cox regression indicated that only severe lung complications increased risk of death in the cohort. There has been a lack of similar studies addressing long term mortality according to severity of late complications. However, this result indicated that when subjects were categorized according to severity of late complications, only severe complications, especially severe lung complications, affected all-cause mortality. Various studies have shown that the severity of the complications, especially lung ones in people exposed to MG, can become more severe over time [15, 16]. Even the evidence has shown that people who were exposed to low doses of MG and did not have acute clinical symptoms such as blisters also developed pulmonary bronchiolitis complications over time [17]. Therefore, preventing the progression of respiratory complications to severe complications in people affected by MG using early detection is very important and can improve the survival of the injured individuals.

In this study, we also investigated the causes of deaths in this population and compared it with Iranian national population using cause-specific SMR. It is necessary to mention that no autopsy was performed to determine the cause of death because of ethical consideration. Therefore, unfortunately, in this study we did not have the cause of almost 45% of deaths. Nevertheless, the results showed that in the case of respiratory diseases, unlike other diseases, the observed death was higher than expected. It is well known that pulmonary injury is the principal cause of mortality in the first few days to weeks after intense exposure to MG. Also, the existence evidences indicate a causal relation between both acute and chronic exposure to sufficient concentrations of MG and long-term mortality due to malignant respiratory and none-malignant respiratory disease, especially bronchitis which is consistent with our result [4, 8, 18]. Of course, it is important to note that despite the evidences for a relation between exposure and long-term mortality of malignant respiratory disease, chronic exposure has been shown to be carcinogenic, while there is still doubt about carcinogenicity of acute exposure. In other words, studies which have reported an association between acute exposure and mortality from malignant respiratory disease, did not interpret this exposure as carcinogenic, but reported its association with increased risk of other lung diseases, such as bronchitis led to cancer. In the present study, we also observed excess death only due to non-malignant respiratory diseases [4, 8, 19]. Therefore, considering the above, increased mortality of respiratory diseases can be justified. However, despite increasing mortalityof respiratory disease, all-cause mortality rate has not increased. In fact, this was partly due to  the greater distribution of mild to moderate lung complications. As we have shown, only severe lung complications can affect all-cause mortality, while there were a low percent of the subjects with severe lung complications in Sardasht; therefore, all-cause mortality was not affected. It should be noted that low percentage of severe lung cases may be partly due to special care. Moreover, special care has reduced diseases and disorders in other organs such as the circulatory system which almost is the leading cause of death, thereby reducing overall mortality. As existing evidence has shown, even in people with lung involvement such as COPD and asthma, circulatory disease is the leading cause of death, and an increase in respiratory deaths without an increase in circulatory system-realted deaths could rarely affect total deaths [20]. Moreover, another reason is that deaths due to some cause in cohort group are far lower than the general population of the country, while receiving special services do not play a role in reducing them. For example, road injury death, which is almost among the top 3 causes of death in the general population of Iran, was very low in these people. Because these people travel very little given their circumstances, and therefore they have fewer deaths due to  road injury  than the general population of the country, which has definitely affected their overall mortality rate. Therefore, in this study, exposure to MG, despite involvement of the lungs and increased respiratory mortality rate, has not increased all-cause mortality rate due to the control of circulatory disease and other leading causes of death.

One of the strengths of this study is its high sample size. It can also be noted that this is the first study to investigate the effects of acute MG exposure in a population with similar characteristics to the general population. In addition, the other strength of this study is the duration of the study, which individuals were included approximately 32 years after the exposure time. The limitations of the study included the lack of information on some variables such as underlying diseases and genotypes of individuals because we used available data. Also, another limitation was the failure to acquire data of all the survivors exposed to MG in Sardasht; therefore, we used only the information of people with valid evidence about their exposure. In other words, their exposure to MG was confirmed by VMAF. We considered them as a sample of all the exposed people, and therefore expressed the results with a confidence interval. Another limitation was that we were not allowed to use autopsy to determine the cause of death; consequently, the cause of death in approximately 45% of deaths were unknown. Nevertheless, it should be noted that even if all the death causes were known, all-cause mortality rate would definitely be lower than expected, and the respiratory mortality would certainly be higher than expected.

While this study is the first one to document long-term mortality rate among the exposed Sardasht population, it is worth noting that previous studies have documented many possible long-term effects of MG exposure on fertility, quality of life, immunological parameter, mental disorders, late complications in various organs, and the incidence of cancer in Sardasht and other parts of Iran. To acquire more information, we can refer to those studies [3, 7, 16, 21–24]. For example, based on results of two systematic reviews, late dermatological, respiratory and ophthalmological complications were the most prevalent complications caused by MG exposure in Iran. Late dermatological complications were mainly chronic severe itching, scars, dry skin, and skin discoloration. Ophthalmological complications included pinguecula, pterygium, chronic conjunctivitis, vascular tortuosity, limbal ischemia, thin corneas, diffuse corneal opacity, dry eye, and keratitis and their clinical symptoms. Respiratory complications were mainly obstructive chronic bronchitis, bronchiolitis, bronchiectasis, asthma, and fibrosis [24, 25]. In addition, some other studies have documented the effects of various treatments used for these people to improve their condition, and these studies are also available for more information [26, 27].

## Conclusion

Overall, this study shows that people exposed to MG had an increased mortality rate due to respiratory diseases after 32 years, which is a specific consequence of the chemical injuries to the respiratory system. However, all-cause mortality rates did not increase over this period. This indicates the impact of special care measures on these individuals immediately after exposure which prevented disease and disorder specifically in circulatory system, and consequently reduced total number of death in long-term. Indeed, this result indicates that acute exposure to MG, even without wearing protective clothing and masks, has not increased all-cause mortality rate after 32 years if accompanied by special and ongoing care for those exposed. This study suggests that special and ongoing care may prevent increase of long-term mortality as a consequence of exposure to a chemical agent and  respiratory complications.

## Data Availability

The dataset supporting the conclusions of this article is available in the Veterans and Martyr Affair Foundation of Iran. Restrictions apply to the availability of the data from VMAF of Iran, which were used under license for the current study, and so are not publicly available. All our analyses were performed within strict privacy rules; that is, only researchers who received a signed permit were allowed to do analyses within a secured environment.

## Abbreviations

MG: Mustard Gas
SMR: Standard Mortality Ratio
VMAF: Veterans and Martyr Affair Foundation
FEV_1_: Forced Expiratory Volume in the First Second
HR: Hazard Ratio
COPD: Chronic Obstructive Pulmonary Disease
